# Evaluation of Inter-Observer Reliability of Animal Welfare Indicators: Which Is the Best Index to Use?

**DOI:** 10.3390/ani11051445

**Published:** 2021-05-18

**Authors:** Mauro Giammarino, Silvana Mattiello, Monica Battini, Piero Quatto, Luca Maria Battaglini, Ana C. L. Vieira, George Stilwell, Manuela Renna

**Affiliations:** 1 Department of Prevention, Asl TO3, Veterinary Service, Area Animal Sanity, 10045 Piossasco, Italy; mgiammarino@aslto3.piemonte.it; 2 Department of Agricultural and Environmental Sciences—Production, Landscape, Agroenergy, University of Milan, 20133 Milan, Italy; silvana.mattiello@unimi.it (S.M.); monica.battini@unimi.it (M.B.); 3 Department of Economics, Management and Statistics, University of Milan-Bicocca, 20126 Milan, Italy; piero.quatto@unimib.it; 4 Department of Agricultural, Forest and Food Sciences, University of Turin, 10095 Grugliasco, Italy; luca.battaglini@unito.it; 5 Centre for Management Studies of Instituto Superior Técnico (CEG-IST), University of Lisbon, 1049-001 Lisbon, Portugal; ana.lopes.vieira@tecnico.ulisboa.pt; 6 Department of Veterinary Medicine, University of Lisbon, 1300-477 Lisbon, Portugal; stilwell@fmv.ulisboa.pt; 7 Department of Veterinary Sciences, University of Turin, 10095 Grugliasco, Italy

**Keywords:** agreement index, animal-based measure, dichotomous categorical indicator, inter-rater reliability

## Abstract

**Simple Summary:**

In order to be effective, on-farm welfare assessment protocols should always rely on reliable, as well as valid and feasible, indicators. Inter-observer reliability refers to the extent to which two or more observers are observing and recording data in the same way. The present study focuses on the problem of assessing inter-observer reliability in the case of dichotomous (e.g., yes/no) welfare indicators and the presence of two observers, in order to decide about the inclusion of indicators in welfare assessment protocols. We compared the performance of the most popular currently available agreement indexes. Some widely used indexes showed their inappropriateness to evaluate the inter-observer reliability when the agreement between observers was high. Other less used indexes, such as Bangdiwala’s B or Gwet’s $\gamma \left(AC_{1}\right)$, were found to perform better and are therefore suggested to assess the inter-observer reliability of dichotomous indicators.

**Abstract:**

This study focuses on the problem of assessing inter-observer reliability (IOR) in the case of dichotomous categorical animal-based welfare indicators and the presence of two observers. Based on observations obtained from Animal Welfare Indicators (AWIN) project surveys conducted on nine dairy goat farms, and using udder asymmetry as an indicator, we compared the performance of the most popular agreement indexes available in the literature: Scott’s π, Cohen’s k, $k_{PABAK}$, Holsti’s H, Krippendorff’s α, Hubert’s Γ, Janson and Vegelius’ J, Bangdiwala’s B, Andrés and Marzo’s ∆, and Gwet’s $\gamma \left(AC_{1}\right)$. Confidence intervals were calculated using closed formulas of variance estimates for π, k, $k_{PABAK,}$ H, α, Γ, J, ∆, and $\gamma \left(AC_{1}\right)$, while the bootstrap and exact bootstrap methods were used for all the indexes. All the indexes and closed formulas of variance estimates were calculated using Microsoft Excel. The bootstrap method was performed with R software, while the exact bootstrap method was performed with SAS software. k, π, and α exhibited a paradoxical behavior, showing unacceptably low values even in the presence of very high concordance rates. B and $\gamma \left(AC_{1}\right)$ showed values very close to the concordance rate, independently of its value. Both bootstrap and exact bootstrap methods turned out to be simpler compared to the implementation of closed variance formulas and provided effective confidence intervals for all the considered indexes. The best approach for measuring IOR in these cases is the use of B or $\gamma \left(AC_{1}\right)$, with bootstrap or exact bootstrap methods for confidence interval calculation.

## 1. Introduction

Animal-based indicators for the assessment of animal welfare need to meet three essential requirements: validity, feasibility, and reliability [1]. The concept of reliability is closely related to the concept of assessment reproducibility, whether the same observer expresses a measure at different times (intra-observer reliability) or whether there are multiple observers to express the measure at the same moment (inter-observer reliability, IOR). The IOR is a fundamental attribute for reliable welfare assessments, especially when the evaluation is carried out using animal-based indicators, which may be associated with a certain level of subjectivity, and biased by the assessors’ previous experience and level of empathy with the animals [2]. However, in animal welfare and behavioral studies, IOR is frequently neglected due to different reasons (e.g., debate on the particular type of statistic to be used, difficulties in involving multiple observers) [3].

While the term “agreement” means the measure of concordance between observers (concordance rate, $P_{o}$), “reliability” is what we would like to infer from the agreement [4]. Reliability measures the concordance between observers, net of chance agreement [5]. If reliability is low, the indicator is inappropriate and should be redefined, ensuring good data recording and/or better training of the observers [6]. The reliability of animal welfare indicators should be bias-free and, furthermore, the indexes should be robust. The reproducibility is the most important interpretation of reliability [4], and it is necessary that the agreement estimates can ensure the reproducibility of judgments. The need to ascertain the agreement between observers, beyond the agreement due to chance, implies the possibility of having reliable statistical methods for assessing the quality of measurements [7].

According to Krippendorff [4], an agreement coefficient can become an index of reliability only if: (i) it is applied to proper reliable data, (ii) it treats units of analysis as separately describable or categorizable without presuming any knowledge about the correctness of their descriptions or categories (absence of gold standard), and (iii) its values correlate with the conditions under which one is willing to rely on imperfect data.

To our knowledge, only a few studies have been specifically designed to test the IOR of animal welfare indicators [8,9,10,11,12,13,14]. For this purpose, the most frequently used agreement index has been Cohen’s k [5]. Some recent reviews, aimed at identifying promising indicators for welfare assessments in ruminants, confirmed that reliability, and particularly IOR, has been scarcely investigated so far [1,15,16], and highlighted the need for further investigation of this issue. One explanation could be that, although the literature is rich in agreement indexes, the problem of finding the best one for different application contexts has not yet been solved [17]. According to Ato et al. [18], all agreement indexes for categorical data can be traced back to three distinct approaches. The first approach, the most widely used in the literature, dates back to Scott’s intuition [19] of having to correct the agreement, described as a percentage of concordant cases out of the total number of observed cases, by eliminating the concordance due to chance. The π index [19], the σ index [20], the k index [5], and the $\gamma \left(AC_{1}\right)$ index [21] belong to this approach. Loglinear modeling is the second approach, which aims to analyze agreement and disagreement patterns by accounting for the discrepancies between the data and expected values, under the hypothesis of independence [22]. Loglinear models generalize to a mixture model by including an unobserved categorical latent variable [18]. In such models, the population is composed of two subpopulations (latent classes): the subpopulation of objects easy to classify by both observers (systematic agreement) and the subpopulation of objects difficult to classify (random agreement and disagreement). This approach allowed Aickin [23] to define a new measure of agreement called the α-coefficient. The third approach is inspired by the traditional multiple-choice test, which allowed Andrés and Marzo [24] to define the ∆ index, under the assumption that each observer can choose only one of *N* possible answers for each object to be evaluated.

The aims of this study are to compare the most popular agreement indexes, as to ascertain the best practice for measuring the agreement between two observers, and to calculate the related confidence intervals when evaluating dichotomous categorical animal-based welfare indicators. To do so, we selected one dichotomous animal-based indicator from the Animal Welfare Indicators (AWIN) welfare assessment protocol for goats [25,26], namely the udder asymmetry, and we used it as an example to test the performance of the different considered agreement indexes.

## 2. Materials and Methods

### 2.1. Dataset

The AWIN protocol for welfare assessment [25,26] was applied by two observers in 10 Italian intensive (AWIN prototype protocol applied from Feb to Jun 2014 [12]), 10 Portuguese intensive (AWIN prototype protocol applied from Jan to Apr 2014 [12]), and 13 Italian extensive (AWIN protocol adapted to extensive conditions applied from Apr to Jul 2019; unpublished data) dairy goat farms. Both assessors were students of the second year of the MSc in Animal Science at the University of Turin (Grugliasco, Italy). Assessor A also had an MSc in Veterinary Science and in Biostatistics, had worked as a veterinarian in the Public Health Service, and had more than 10 years of experience with dairy goats. Assessor B had no specific experience with dairy goats. Before the beginning of the study, both assessors received a common 1-day training including both theoretical and practical sessions, and received the AWIN protocol [25] as training material. The training was provided by two authors of the AWIN welfare assessment protocol for goats kept in intensive or semi-intensive production systems [26].

The collected data were preliminarily analyzed to identify a dichotomous variable that presented a wide variation of concordance rate between two observers. Among the six dichotomous categorical individual animal-based welfare indicators included in the AWIN protocol for goats (i.e., fecal soiling, nasal discharge, ocular discharge, severe lameness, overgrown claws, and udder asymmetry), udder asymmetry was chosen to test different methods for assessing IOR, because it was the variable where we observed the widest variability of agreement between observers in the visited farms. According to the AWIN protocol, during the assessment, each goat was assigned to one of two mutually exclusive and exhaustive categories: presence of asymmetry = 1; absence of asymmetry = 0. The presence of udder asymmetry was confirmed when one half of the udder was at least 25% longer than the other, excluding the teats [26].

To perform our analysis, we used data collected from nine farms (out of the initially considered 33 farms), which represented the whole range of variability (75 to 100%) in terms of agreement between observers: seven Italian intensive farms (from I-IT1 to I-IT7), one Portuguese intensive farm (I-PT1), and one Italian extensive farm (E-IT1).

### 2.2. Agreement Measures

A crude measure of reliability ($P_{o}$) is given by the proportion of concordant cases (agreement) out of the total observed cases. However, this measure is distorted in favor of situations with fewer categories [19] and does not account for the chance agreement.

The IOR of udder asymmetry was evaluated measuring the most popular agreement indexes currently available in the literature: Scott’s π [19], Cohen’s k [5], $k_{PABAK}$ and the related indexes (σ [20], G [27], and S [28]), Holsti’s H [29], Krippendorff’s α [30], Hubert’s Γ [31], Janson and Vegelius’ J [32], Bangdiwala’s B [33], Andrés and Marzo’s ∆ [24], and Gwet’s $\gamma \left(AC_{1}\right)$ [21]. A detailed description of each considered agreement index is presented in Appendix A.

### 2.3. Confidence Intervals for Agreement Indexes

Closed formulas of variance estimates are available for almost all the considered agreement indexes. The application of such formulas is handy for some indexes, including π [19], k and $k_{C}$ [5], $k_{PABAK}$ and the related indexes (σ [20], G [27], and S [28]), Holsti’s H [29], α [30], Γ [31], J [32], ∆ [24], and $\gamma \left(AC_{1}\right)$ [21]. A detailed description of the applied formulas of variance estimates for the above-mentioned indexes is presented in Appendix B.

Closed formulas of variance estimates are instead cumbersome for the *B* index. Therefore, for such index, we used confidence intervals based on the bootstrap method [34] and the exact bootstrap method for small samples [35]. Bootstrapping is a general method for estimating the distribution of a given statistic by resampling with the replacement of the data set at hand [34]. The bootstrap procedure uses such an empirical distribution as a substitute for the true distribution in order to provide variance estimates and confidence intervals. A criticism of the standard bootstrap procedure is that different observers may reach, by chance, different conclusions [35]. The exact bootstrap method prevents the possibility of different conclusions. This method was proposed for Cohen’s k when the proportion of agreement was high, and the sample size *n* was small (≤200), but it was never applied to other agreement indexes so far. The exact bootstrap method attributes the probability 1/*n* to each element of a small population with size *n*, so that we can extract with replacement *n*^*n* samples from the population, which allows providing *n*^*n* values of the considered agreement index, whose empirical distribution is known as the exact bootstrap distribution [35]. In particular, 95% bootstrap and exact bootstrap confidence intervals can be constructed by the percentile method, which employs the 2.5th and 97.5th percentiles of the bootstrap and exact bootstrap distribution, respectively [35,36].

### 2.4. Statistical Analyses

Microsoft Excel (2010) was used to calculate the index values (using the formulas reported in Appendix A) and their confidence intervals (using the closed formulas of variance estimates reported in Appendix B). For bootstrapping, the following packages of the R software (v. 3.5.2; R Core Team, Wien, Austria, 2018) were used: “raters” [37], “vcd” [38] and “bootstrap” [39]. The SAS software (v. 9.0; SAS Institute Inc., Cary, NC, USA) was used for the exact bootstrap method, using the script reported by Klar et al. [35] for Cohen’s k; the scripts were modified by adapting them to the all the other considered agreement indexes.

## 3. Results

### 3.1. Agreement Measures

Three hundred and eighty-eight dairy goats were examined in the nine selected farms. The frequency of cases for the indicator “udder asymmetry” in each of the nine selected farms is reported in the agreement tables (Table S1).

For each farm, Table 1 shows the values expressed by the considered agreement indexes for the AWIN indicator “udder asymmetry”.

As expected, the H index coincided with the concordance rate ($P_{o}$). The k index and α index on the one hand, and the Γ index and J index on the other hand, showed the same values. The π index, k index, and α index expressed unacceptably low values, even in the presence of high concordance rates (e.g., farms I-IT2, I-IT5, and I-IT7). When the concordance between observers was perfect, and cell *n11* of the agreement table (Table S2) showed a value equal to zero, π index, k index, and α index did not express any value. When the concordance was not perfect for a single or few objects, and cell *n22* showed a value equal to zero (farms I-IT5 and I-IT7), Cohen’s k and Scott’s π showed value zero or a negative value since one of the marginals relating to the probability table was zero.

The distance from the concordance rate of the values expressed by the $k_{PABAK}$ index (the values of which coincided with those of the related σ, G, and S indexes) gradually decreased as the concordance rate increased (Table 1) until it expressed the value 1 to perfect concordance.

The ∆ index showed an intermediate behavior between the $k_{PABAK}$, with which it shared the values when the concordance rate ranged from 75 to 92%, and the B index, the values of which were similar to those expressed by the ∆ index at higher concordance rates (95 to 100%). The distances between the values expressed by the ∆ index and the concordance rate were wider at medium-high values of concordance rate (75 to 92%), but soon they decreased, and ∆ index coincided with the concordance rate in the case of higher concordance rates (95 to 100%; Table 1).

The Γ index expressed low values especially when the concordance rate was equal to 75 and 77% (farms E-IT1 and I-IT1). The distances from the values of $P_{o}$ were high, up to values of 97% of the concordance rate (Table 1).

The B index showed values very close to those of the concordance rate in all the cases examined in this study. When the concordance rate showed its minimum (75%; farm E-IT1), the B index showed the highest value among the values presented by the analyzed indexes (Table 1). The B index values were always very close to those of the observed concordance rate until they early coincided with them (when $P_{o}$ = 88%, B index = 0.87; farm I-IT2). The Bangdiwala’s observer agreement chart (Table S1) graphically represents the B index, providing an immediate and very useful visual representation of the obtainable results.

The $\gamma \left(AC_{1}\right)$ index expressed almost the same values of the B index, with the exception of cases with medium-high values of concordance rate (75 and 77%; farms E-IT1 and I-IT1), when the $\gamma \left(AC_{1}\right)$ index showed lower values than the B index.

### 3.2. Confidence Intervals for Agreement Indexes

**Figure 1:**
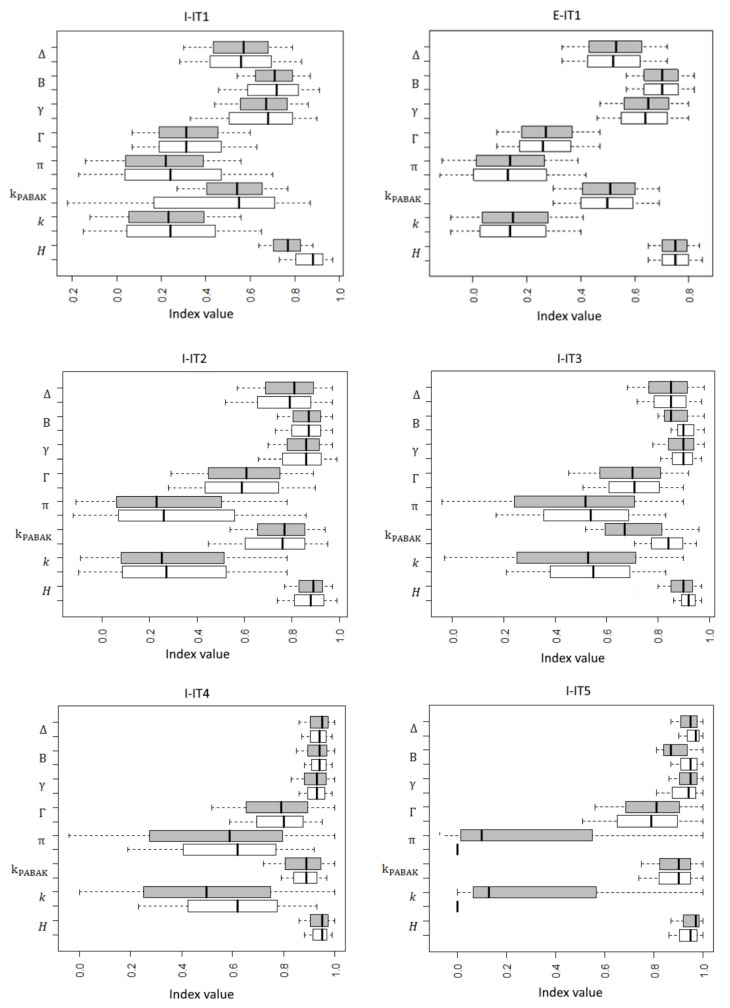

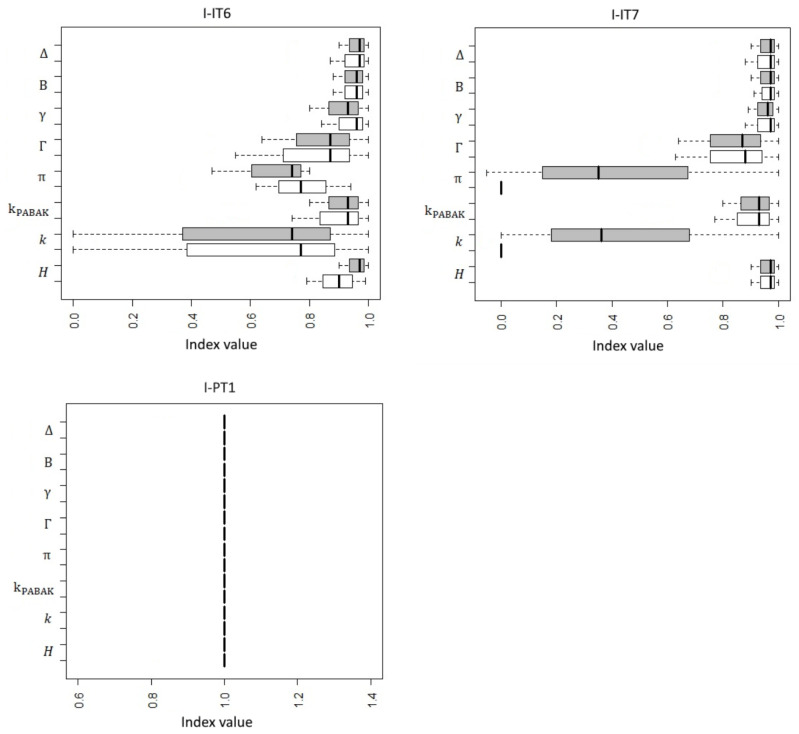
Boxplot of the agreement values obtained for each index with the bootstrap method and the exact bootstrap method for all the selected farms (I-IT1, E-IT1, I-IT2, I-IT3, I_IT4, I_IT5, I_IT6, I-IT7, I-PT1). Legend: ∆ = ∆ index; B = B index; γ = $\gamma \left(AC_{1}\right)$ index; Γ = Γ index; π = π index; $k_{\mathrm{PABAK}}$ coincided with the related indexes: σ index, G index and S index; *k* = k index; *H* = H index; grey = bootstrap method; white = exact bootstrap method. The α index is not reported in the figure as it coincided with Cohen’s k. The J index is not reported in the figure as it coincided with Hubert’s Γ.

Figure 1 shows the boxplot of the values obtained for each considered agreement index with the bootstrap method and the exact bootstrap method for the nine selected farms.

The best performing indexes are expressed by values closer to the concordance rate (that coincided with Holsti’s H) and by narrower confidence intervals. For all the considered indexes and for all the farms, we observed a substantial overlapping of confidence intervals results when implemented with the bootstrap and exact bootstrap methods. The inadequacy of the values expressed by Cohen’s k and Scott’s π is evident in the case of low concordance rates (farms E-IT1, I-ITI, I-IT2, I-IT5, and I-IT7). In all the cases, confidence intervals ranges were wide for π and k indexes, even when no paradox effect was observed. In almost all cases, the Γ e $k_{PABAK}$ indexes also showed wider ranges of confidence intervals when compared to the other considered agreement indexes. The exact bootstrap method expressed confidence intervals for π and k indexes even when cell *n22* of the agreement table showed a value equal to zero (Figure 1, boxplots for farms I-IT5 and I-IT7). The boxplots also graphically highlight the paradox effect (farms E-IT1, I-IT1 and I-IT2).

For each index, confidence intervals calculated using closed formulas of variance estimates and both the bootstrap and exact bootstrap methods are presented for the nine selected dairy goat farms in Table S1.

## 4. Discussion

From the results obtained in our study, it is evident that, when evaluating IOR, the choice of the agreement index is very subtle and becomes crucial in order to validate the method of evaluating welfare indicators. The paradoxical behavior of Cohen’s k, Scott’s π, and Krippendorff’s α makes it difficult to use these indexes without a careful critical analysis of the results. For this reason, it is recommended to use other indexes that are not affected by the same paradox effect. For the evaluation of IOR in the case of dichotomous categorical indicators and the presence of two observers, Bangdiwala’s B and Gwet’s $\gamma \left(AC_{1}\right)$ were found to be the most appropriate indexes to be used.

When trying to find an adequate approach to evaluate the IOR of animal-based welfare indicators, it is very common to get lost within the array of different concepts and methods. Furthermore, it is common to find criticisms of different order for each method, which makes the selection even more difficult. In this work, we aimed to clear this on-going discussion by focusing on dichotomous categorical animal-based welfare indicators in the presence of two observers. The literature shows the limitations of the method of calculating the agreement between observers by the proportion of concordant cases out of the total cases, without taking into account the concordance due to chance [40]. The same criticism involves the H index that, as expected, was unable to calculate the agreement by chance [41].

To evaluate the IOR, some authors used the approach based on the χ^2^ test, calculated from a cross-classification table, or the approach based on correlation coefficients. However, both approaches appear unsuitable and, consequently, they were not implemented in this study. The χ^2^ test measures the degree of independence between variables that does not necessarily coincide with concordance. In fact, the association measures calculate the deviation from chance contingencies between variables [4]. Therefore, the χ^2^ statistic presents high values for any deviation from the association due to chance, both in case of agreement and in case of disagreement [40]. Similarly, the use of correlation coefficients that measure deviations from linearity is also discouraged because correlation and concordance are not the same [42]. According to Krippendorff [4], a valid index measures agreements or disagreements among multiple descriptions generated by a single coding procedure, regardless of who enacts the procedure.

Cohen [5] proposed the k index as an extension of Scott’s π [19], which in defining the rate of agreement due to chance involves the knowledge of rate distributions for both observers. It assumes that the two observers are interchangeable, so that the marginal distributions are identical and hence the two indexes of Cohen and Scott are equivalent [40]. Although the k index is still the most widely used agreement index [43], in some circumstances where the concordance rate is very high, it shows unacceptably low values. Such a paradoxical behavior of Cohen’s k is well studied in the literature [44,45]. To overcome this problem, Byrt et al. [46] proposed two diagnostics for k given by $BI=\left(n_{12}-n_{21}\right)/N$ (bias index) and $PI=\left(n_{11}-n_{22}\right)/N$ (prevalence index): BI is zero when the marginal distributions are equal and PI is zero when the categories are equally likely [47]. However, all this would make the reading of the value less immediate and the interpretation of the index more difficult. This is the reason why Byrt’s diagnostics were not implemented in our study. Our results confirm the paradoxical behavior of the k index, as it showed unacceptably low values even in the presence of very high concordance rates. Landis and Koch [48] suggested different ranges of values for the k index: values higher than 0.74 indicate excellent agreement; values between 0.40 and 0.75 indicate a good agreement; and values less than 0.40 indicate a poor agreement. However, the same authors claimed that every categorization is arbitrary. In Table S1, where the concordance rate is 75%, k index (0.16) demonstrated a slight agreement according to the benchmarking proposed by Landis and Koch [48] and a marginal agreement according to the benchmarking of Fleiss [49]. This is also evident in Table 1 for farms I-IT1 (k index = 0.24; $P_{o}$ = 77%), I-IT2 (k index = 0.27; $P_{o}$ = 88%), and I-IT3 (k index = 0.55; $P_{o}$ = 92%). Paradoxical behaviors are also evident in Vieira et al. [12], where a concordance rate of 92.42% corresponded to a mediocre value of the k index (0.44). For this reason, the k index cannot be considered adequate to analyse the IOR in the case of dichotomous categorical animal-based welfare indicators (such as the udder asymmetry evaluated in our study), for which the concordance between observers is presumed to be very high, even close to 100% in some cases [12]. More precisely, the paradox of the k index is twofold. The first paradox occurs when the marginal totals are highly unbalanced in a symmetrical way (e.g., farm E-IT1; Table S1), producing high values of $P_{e}$. The second paradox, not observed in our study but reported in the literature, appears when the marginal totals are asymmetrically balanced, producing values which cannot be high [44]. The $k_{M}$ version proposed by Cohen [5] does not seem to avoid the two types of paradox [44]. Cicchetti and Feinstein [50] suggested tackling the paradox by adopting two indexes to account for the two paradoxes. We agree with Brennan and Prediger [51] that the indiscriminate use of the k index can be misleading and that other statistics may be more meaningful in some cases. Other authors [12] tried to overcome this paradox by presenting, simultaneously, information on the overall agreement together with positive and negative agreement, and the prevalence of the indicator. However, even if this presents the reader with all the information for analysis, it puts an extra cognitive burden on whomever is analyzing the data, which can hinder its interpretation. For this reason, further research on the topic that assists in overcoming this drawback is needed.

The α index [30] assumes values very close to the k index [5], as they belong to the same approach. This is also confirmed by the results obtained in the current study, where the two indexes showed exactly the same values for all the nine considered farms. From our results, it seems that the α index suffers from the same paradoxical behavior as Cohen’s k, as previously reported by Zhao [52] and Gwet [53].

From the analysis of our results, it appears evident that also the π index suffers the same paradoxical behavior seen for the k index, which represents an extension of π (see for example farms E-IT1, I-IT1, and I-IT2, where the values of the indexes are very far from $P_{o}$). In an interesting comparative publication of several indexes for 2 × 2 tables [18], both π index and k index produced very high distortions at extreme prevalence values and were shown to be the least well-performing indexes.

The $k_{PABAK}$ does not show the paradox effects [47], as confirmed by the results obtained in our study. In the work of Ato et al. [18], the σ index [20] was considered as an unbiased index that presented an excellent behavior for 2 × 2 tables. The S index [28] also allows measuring the level of inter-rater agreement without incurring the paradoxes of the k index [54]. The G index has reasonably small biases for estimating the “true” IOR [21].

The ∆ index [24] has also proven to be reliable in this study, confirming previous results obtained by Ato et al. [18].

The B index [33] showed the highest value among all considered indexes when the concordance rate attained the minimum value (75%) (farm E-IT1). However, at very high concordance rates, it gave the same values as the ∆ index (farm I-IT7). If only one of the diagonal cells of the agreement table (Table S2) exhibits agreement, the B index equals $P_{o}$. In addition, the Bangdiwala’s observer agreement chart (Table S1) represents an immediate and useful tool that does not suffer from the paradox effect [47] and is easily obtainable with the PROC FREQ of the SAS program [55] or by the “vcd” package of the R program [38].

The $\gamma \left(AC_{1}\right)$ index [21] is recommended [56,57], even if it is not widely adopted [17] because it is little known. In particular, it also equals $P_{o}$ when the concordance is present in only one of the diagonal cells of the agreement table [47].

In order to provide confidence intervals, the bootstrap and exact bootstrap methods turned out to be simpler when compared to the implementation of closed variance formulas and, in particular, the exact bootstrap method is easily executable in SAS [35].

## 5. Conclusions

When evaluating dichotomous categorical animal-based welfare indicators, and particularly in the case of a high concordance rate, the optimal practice for measuring the IOR between two observers is the use of the B index [33] or the $\gamma \left(AC_{1}\right)$ index [21], as they are not affected by paradoxical behaviors. Both the bootstrap and exact bootstrap methods are easier to be executed when compared to closed formula of variance estimates and provide effective confidence intervals for all the considered agreement indexes, including B and $\gamma \left(AC_{1}\right)$. Our study also clearly demonstrates that the exact bootstrap is a valid method for the calculation of confidence intervals not only for the π index and k index, as already reported in the published literature, but for all the tested agreement indexes.

Our results can be extended to any welfare assessment protocol (e.g., other species or different contexts of application) when two independent observers test dichotomous variables at the same time. Further studies are needed to find the best practice to assess IOR for other types of variables (e.g., trichotomic and four-level variables), also in the presence of more than two observers.

## Figures and Tables

**Table 1:** Values of the agreement indexes for the AWIN animal-based welfare indicator “udder asymmetry” for the nine selected dairy goat farms, sorted by increasing concordance rate ($P_{0}$).

		Agreement Index ^1^										
Farm	$P_{o}$ ^2^	π	k	$k_{c}$	$k_{PABAK}$ ^3^	H	α	Γ	J	B	∆	$\gamma \left(AC_{1}\right)$
E-IT1	75	0.15	0.16	0.23	0.51	75	0.15	0.25	0.25	0.70	0.52	0.65
I-IT1	77	0.24	0.24	0.24	0.54	77	0.24	0.28	0.30	0.71	0.54	0.68
I-IT2	88	0.27	0.27	0.43	0.77	88	0.28	0.58	0.58	0.87	0.79	0.86
I-IT3	92	0.55	0.55	0.55	0.84	92	0.56	0.69	0.70	0.90	0.84	0.90
I-IT4	95	0.64	0.64	1.00	0.89	95	0.64	0.79	0.79	0.94	0.95	0.94
I-IT5	95	−0.02	0.00	0.00	0.90	95	−0.01	0.80	0.81	0.95	0.95	0.95
I-IT6	97	0.78	0.78	1.00	0.93	97	0.78	0.87	0.87	0.96	0.97	0.96
I-IT7	97	−0.02	0.00	0.00	0.93	97	0.00	0.87	0.87	0.97	0.97	0.96
I-PT1	100	1.00	1.00	1.00	1.00	100	1.00	1.00	1.00	1.00	1.00	1.00

Abbreviations: E, extensive; I, intensive; IT, Italian; PT, Portuguese. ^1^ π [19]; k [5]; $k_{c}$ [5]; H [29]; α [30]; Γ [31]; J [32]; B [33]; ∆ [24]; $\gamma \left(AC_{1}\right)$ [21]. ^2^ Concordance rate ($P_{o}$, %), calculated as: $\left(n_{11}+n_{22}\right)/N$. ^3^ The related indexes (σ index [20], G index [27], and S index [28]) gave the same results.

## Data Availability

The data presented in this study are available on request from the corresponding author.

## Supplementary Materials

The following are available online at https://www.mdpi.com/article/10.3390/ani11051445/s1, Table S1: Motivation examples, Table S2: Agreement table.

## Appendix A

The considered agreement indexes are presented here in the chronological order by which they were developed.

### Appendix A.1. π Index

(A1) $$\pi =\frac{P_{o}-P_{e}}{1-P_{e}}$$

where [19]:

$P_{o}$ is the rate of observed concordance and represents the rate of concordant judgments of two independent observers who analyze the same dataset;
$P_{e}$ is the rate of the expected agreement due to chance given by:
  (A2) $$P_{e}=\overset{M}{\underset{i=1}{\sum }}p_{i}^{2}$$
  where:
  *M* is the number of categories;
  $p_{i}$ is the proportion of objects assigned to the *i*-th category.

This index varies from 0 (no agreement) to +1 (perfect agreement).

### Appendix A.2. k and $k_{C}$ Indexes

(A3) $$k=\frac{\sum P_{ii}-\sum P_{i.}P_{.i}}{1-\sum P_{i.}P_{.i}}$$

where [5]:

$\sum P_{ii}$ is the observed hit rate, denoted by $P_{o}$;
$\sum P_{i.}P_{.i}$ is the proportion of agreement due to chance, denoted by $P_{e}$. Hence, the formula can be summarized as:
  (A4) $$k=\frac{P_{o}-P_{e}}{1-P_{e}}$$
  The assumptions for k are the following [5,51]:
  (a) The *N* objects categorized are independent;
  (b) The categories are independent, mutually exclusive, and exhaustive;
  (c) The assigners operate independently.

By examining the formula, it can be seen that Cohen [5] standardizes the difference between the observed agreement and the expected agreement, dividing it by the difference between the maximum value of k and the amount of agreement due to chance. In general, k assumes values between $-P_{e}/\left(1-P_{e}\right)$ and 1. The maximum value is reached only if the values outside the diagonal of the agreement table (Table S2) are zero and the marginal totals of the two observers are equal. However, when the marginal totals are asymmetric (as it happens very often), the maximum value of k will never be 1. To deal with this issue, Cohen [5] suggested the k maximum value:

(A5) $$k_{M}=\frac{P_{oM}-P_{e}}{1-P_{e}}$$

where:

$P_{oM}$ is the maximum observed proportion, obtained by adding the minimum values of the individual marginal totals.

Cohen [5] estimated the k correct ($k_{C}$) dividing k by *k_M_*:

(A6) $$k_{C}=\frac{k}{k_{M}}$$

These indexes vary from 0 (no agreement) to +1 (perfect agreement).

### Appendix A.3. $k_{PABAK}$

Many authors proposed an adjusted *k* given by:

(A7) $$k_{PABAK}=2*P_{o}-1\;$$

where:

$P_{o}$ is the concordance rate.

Despite being characterized by different formulas, the σ index [20], the G index [27], and the S index [28] are traced back to this criterion of correction.

This index varies from −1 (no agreement) to +1 (perfect agreement).

### Appendix A.4. H Index

(A8) $$H=P_{o}=\frac{2*C}{N_{A}+N_{B}}$$

where [29]:

*C* is the number of concordant judgments;
*N_A_* is the number of judgments of the observer A;
*N_B_* is the number of judgments of the observer B.

This index is expressed as a percentage and varies from 0 (no agreement) to 100% (perfect agreement).

### Appendix A.5. α Index

The index aggregates disagreements observed on individual units and compares such an observed agreement to the agreement expected by chance:

(A9) $$\alpha =\frac{P_{o}-P_{e}}{1-P_{e}}$$

where [30]:

(A10) $$P_{o}=\left(n_{11}+n_{22}\right)/n$$

while

(A11) $${\hat{P}}_{e}=\left(\frac{2m_{1}}{2n}\right)\left(\frac{2m_{1}-1}{2n-1}\right)+\left(\frac{2m_{2}}{2n}\right)\left(\frac{2m_{2}-1}{2n-1}\right)$$

with

(A12) $$m_{1}=\frac{n_{+1}+n_{1+}}{2}\;\mathrm{and}\;m_{2}=\frac{n_{+2}+n_{2+}}{2}$$

This index varies from 0 (no agreement) to +1 (perfect agreement).

### Appendix A.6. Γ Index

This index [29] was proposed as a particular case of Kendall’s generalized correlation coefficient [32,58]:

(A13) $$\Gamma =1-4*\frac{\left(n_{11}+n_{22}\right)\left(n_{12}+n_{21}\right)}{n\left(n-1\right)}$$

When all the cells are equal, this index does not satisfy the reasonable requirement of being null [32].

This index varies from 0 (no agreement) to +1 (perfect agreement).

### Appendix A.7. J Index

This index [32] was introduced as a correlation coefficient for nominal scale and represents a particular case of Kendall’s general coefficient [58]:

(A14) $$J=\frac{{\left[\left(n_{11}+n_{22}\right)-\left(n_{12}+n_{12}\right)\right]}^{2}}{n^{2}}$$

The J index is closely related to Hubert’s Γ, but it does not have the problem above-mentioned for *Γ* [59].

This index varies from 0 (no agreement) to +1 (perfect agreement).

### Appendix A.8. B Index

The B index [33] summarizes the so-called “observer agreement chart”, in which the degree of agreement is represented by the proportion of the two dark square areas shown in Table S1:

(A15) $$B=\frac{\sum_{i=1}^{k}a_{ii}^{2}}{\sum_{i=1}^{k}n_{i.}n_{.i}}$$

where:

$a_{ii}^{2}$ is the square of the values of the concordant cells;
$n_{i.}$ is the total of *i*-th row;
$n_{.i}$ is the total of *i*-th column.

Being a proportion of areas, this index also varies from 0 (no agreement) to +1 (perfect agreement) [47].

### Appendix A.9. ∆ Index

Cohen’s k may have a paradoxical behavior when marginal distributions are asymmetric [45]. Andrés and Marzo [24] proposed the so-called ∆ index, which aims to be independent of marginal distributions. For 2 × 2 tables, these authors suggested an asymptotic approximation of the index, which can be used as a consistent measure of concordance [24]:

(A16) $$∆=p_{11}+p_{22}-2\sqrt{p_{12}p_{21}}$$

This index varies from 0 (no agreement) to +1 (perfect agreement).

### Appendix A.10. $\gamma \left(AC_{1}\right)$ Index

To avoid the k paradoxical behavior, Gwet [21] proposed the coefficient of agreement $\gamma \left(AC_{1}\right)$ [21]:

(A17) $$\gamma (AC_{1})=\frac{P_{o}-P_{e}^{*}}{1-P_{e}^{*}}\in \left[-1,1\right]$$

where:

$P_{o}$ is estimated with $P_{o}=(n_{++}+n_{--})/n$;
$P_{e}^{*}$ is estimated as:
  (A18) $$P_{e}^{*}=2\pi_{+}\left(1-\pi_{+}\right)$$
  with
  (A19) $$\begin{array}{c} \pi_{+}=\left(p_{Oss1|0}+p_{Oss2|0}\right)/2,\\ p_{Oss1|0}=n_{Oss1|0}/n\;\mathrm{and}\;p_{Oss2|0}=n_{Oss2|0}/n. \end{array}$$

This index varies from 0 (no agreement) to +1 (perfect agreement).

## Appendix B

Here follows a description of the applied closed formulas of variance estimates.

### Appendix B.1. π Index

Scott [19] proposed the following formula for the variance of π index:

(A20) $$Var\left(\pi \right)={\left(\frac{1}{1-p_{e}}\right)}^{2}\frac{p_{o}\left(1-p_{o}\right)}{n-1}$$

### Appendix B.2. k, $k_{C}$, and α Indexes

In order to determine whether k differs significantly from zero, Fleiss et al. [60] proposed a formula for an asymptotic approximation of the variance in the case of an *m* × *m* table. Under the hypothesis of the agreement occurring by chance, the asymptotic variance equals the exact variance proposed by Everitt [61] based on the hypergeometric distribution:

(A21) $$Var_{0}\left(k\right)=\frac{p_{e}+p_{e}^{2}-\sum_{i=1}^{m}p_{i.}p_{.i}\left(p_{i.}+p_{.i}\right)}{n{\left(1-p_{e}\right)}^{2}}$$

For large *n*, a simplified version of the Fleiss’ formula

(A22) $$Var\left(k\right)=\frac{1}{n{\left(1-p_{e}\right)}^{2}}\left\{\overset{m}{\underset{i=1}{\sum }}p_{ii}{\left[1-\left(p_{i.}+p_{.i}\right)\left(1-\hat{k}\right)\right]}^{2}+{\left(1-\hat{k}\right)}^{2}\overset{m}{\underset{i\neq j}{\sum }}p_{ij}{\left(p_{i.}+p_{.j}\right)}^{2}-{\left[\hat{k}-p_{e}\left(1-\hat{k}\right)\right]}^{2}\right\}$$

is given by Altman et al. [62].

(A23) $$Var\left(k\right)=\frac{{\hat{p}}_{o}\left(1-{\hat{p}}_{o}\right)}{n{\left(1-p_{e}\right)}^{2}}$$

This allowed us to build an asymptotic two-side 1−α confidence interval for *k*:

(A24) $$k\pm \sigma_{\hat{k}}*z_{1-\alpha /2}$$

where $\sigma_{\hat{k}}$ is the standard error of $\hat{k}$ and $z_{1-\alpha /2}$ is the quantile of the standard normal distribution.

The same closed formulas of variance estimates used for k and $k_{C}$ were also implemented for α index, as these indexes belong to the same approach [62].

### Appendix B.3. $k_{PABAK}$ Index

According to Scott [19], we calculated the following formula:

(A25) $$Var\left(k_{PABAK}\right)=4\frac{p_{o}\left(1-p_{o}\right)}{n-1}$$

### Appendix B.4. H Index

Following Scott [19], we obtained the simple formula:

(A26) $$Var\left(H\right)=\frac{p_{o}\left(1-p_{o}\right)}{n-1}$$

### Appendix B.5. J and Γ Indexes

Janson and Vegelius [59] proposed a simple formula for the variance estimation of the J index:

(A27) $$Var\left(J\right)=\frac{2}{n^{2}}$$

The same closed formula of variance estimates used for J was also implemented for Γ index, as these indexes belong to the same approach [59].

### Appendix B.6. ∆ Index

Andrés and Marzo [24] presented these formulas for standard deviation:

(A28) $$SE\left({∆}_{I}\right)=\sqrt{\frac{\left(1-∆\right)\left(1+∆\right)}{n}}$$

(A29) $$SE\left({∆}_{II}\right)=\sqrt{\frac{\left(1-∆\right)}{n}\overset{2}{\underset{i=1}{\sum }}\frac{x_{ii}}{n_{i}}}$$

### Appendix B.7. $\gamma (AC_{1})$ Index

Gwet [21] proposed a complex formula for the variance of $\gamma \left(AC_{1}\right)$ index:

(A30) $$\begin{array}{r} Var\left(\hat{\gamma }\right)=\frac{1-f}{n{\left(1-p_{e}\right)}^{2}}\{p_{o}\left(1-p_{o}\right)-4\left(1-\hat{\gamma }\right)\left(\frac{1}{q-1}\overset{q}{\underset{k=1}{\sum }}p_{kk}\left(1-{\hat{\pi }}_{k}\right)-p_{o}p_{a}\right)\\ +4{\left(1-\hat{\gamma }\right)}^{2}\left(\frac{1}{{\left(q-1\right)}^{2}}\overset{q}{\underset{k=1}{\sum }}\overset{q}{\underset{l=1}{\sum }}p_{kl}{\left[1-\left({\hat{\pi }}_{k}+{\hat{\pi }}_{l}\right)/2\right]}^{2}-p_{e}^{2}\right)\} \end{array}$$
