# Supplementary material for: Evaluation of Inter-Observer Reliability of Animal Welfare Indicators: Which Is the Best Index to Use?
Source: Animals (Basel). 2021 May 18;11(5):1445. doi: 10.3390/ani11051445 (PMC8157558; doi:10.3390/ani11051445)
Supplement: Supplementary file 1 [file animals-11-01445-s001.zip › animals-1177606-s001.pdf]

**Table S1**. Agreement table, observer agreement chart, agreement indexes and relative confidence intervals for the selected nine dairy goat farms.

Values of the agreement indexes used with relative confidence intervals, calculated with the closed formulas of variance estimates found in published literature and through the bootstrap and exact bootstrap methods. The first column shows the agreement table [63] and the observer agreement chart [36].

[1] $\pi$ = $\pi$ index [19]; $k$ = $k$ index [5]; $k_c$ = correct $k$ index [5]; $k_{PABAK}$ and related indexes ($\sigma$ index [20], G index [27] and S index [28]); $H$ = $H$ index [29]; $\alpha$ = $\alpha$ index [30]; $\Gamma$ = $\Gamma$ index [31]; $J$ = $J$ index [32]; $B$ = $B$ index [33]; Δ = Δ index [24]; $\gamma(AC_1)$ = $\gamma(AC_1)$ index [21].

[2] E-IT1, I-IT1, I-IT2, I-IT3, I-IT4, I-IT5, I-IT6, I-IT7, I-PT1 = farms.

[3] $P_o$ = concordance rate.

[4] N.C. = not calculated as the closed formula or the algorithm was not able to give any value.

| | Index[1] | Value | Confidence intervals | |
|---|---|---|---|---|
| | | | By closed formula | By bootstrap method (BM; mean)<br>By exact bootstrap method (EBM; mean) |

E-IT1[2]

| | | A2 | | |
|---|---|---|---|---|
| | | 0 | 1 | |
| A1 | 0 | **54** | **13** | 67 |
| | 1 | **6** | **4** | 10 |
| | | 60 | 17 | 77 |

Agreement table

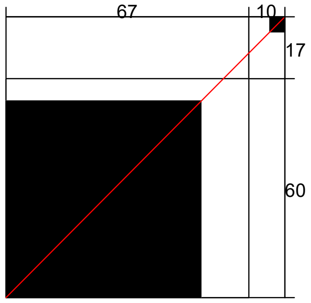


Observer agreement chart

| Index | Value | By closed formula | By bootstrap method (BM; mean) / By exact bootstrap method (EBM; mean) |
|---|---|---|---|
| $P_o$ = 0.75 [3] | | | |
| $\pi$ | 0.15 (with paradoxes) | -0.19; 0.48 [19] | BM: - 0.12; 0.42 (0.13)<br>EBM: - 0.11; 0.39 (0.14) |
| $k$<br>$\alpha$ | 0.16 (with paradoxes) | -0.01; 0.33 [62] | BM: -0.08; 0.40 (0.14)<br>EBM: -0.08; 0.41 (0.15) |
| $k_C$ | 0.23 (with paradoxes) | 0.01; 0.45 [62] | BM: -0.14; 0.62 (0.21)<br>EBM: -0.13; 0.60 (0.22) |
| $k_{PABAK}$ | 0.51 (without paradoxes) | 0.31; 0.69 [19] | BM: 0.30; 0.69 (0.50)<br>EBM: 0.30; 0.69 (0.51) |
| $H$ | 0.75 (without paradoxes) | 0.65; 0.85 [19] | BM: 0.65; 0.85 (0.75)<br>EBM: 0.65; 0.84 (0.75) |
| $\Gamma$<br>$J$ | 0.25 (with paradoxes) | 0.22; 0.29 [59] | BM: 0.09; 0.47 (0.26)<br>EBM: 0.09; 0.47 (0.27) |
| $B$ | 0.70 (without paradoxes) | N.C.[4] | BM: 0.57; 0.82 (0.70)<br>EBM: 0.57; 0.82 (0.70) |
| $\Delta$ | 0.52 (without paradoxes) | $\Delta_I$= 0.33; 0.71<br>$\Delta_{II}$ = 0.35; 0.69<br>[24] | BM: 0.33; 0.72 (0.52)<br>EBM: 0.33; 0.72 (0.53) |
| $\gamma$ ($AC_1$) | 0.65 (without paradoxes) | 0.54; 0.77 [21] | BM: 0.46; 0.80 (0.64)<br>EBM: 0.47; 0.80 (0.65) |

I-IT1

| | | A2 | | |
|---|---|---|---|---|
| | | 0 | 1 | |
| A1 | 0 | **31** | **5** | 36 |
| | 1 | **5** | **3** | 8 |
| | | 36 | 8 | 44 |

Agreement table

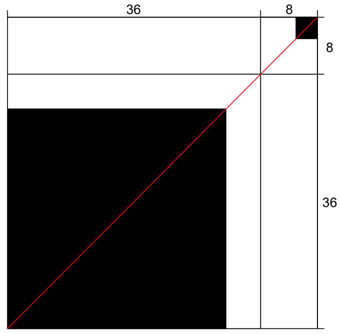


Observer agreement chart

| Index | Value | By closed formula | By bootstrap method (BM; mean) / By exact bootstrap method (EBM; mean) |
|---|---|---|---|
| $P_o$ = 0.77 | | | |
| $\pi$ | 0.24 (with paradoxes) | -0.18; 0.65 [19] | BM: -0.17; 0.70 (0.24)<br>EBM: -0.14; 0.56 (0.22) |
| $k$<br>$\alpha$ | 0.24 (with paradoxes) | -0.18; 0.65 [62] | BM: -0.15; 0.65 (0.24)<br>EBM: -0.12; 0.56 (0.23) |
| $k_C$ | 0.24 (with paradoxes) | 0.24; 0.24 [62] | BM: -0.26; 0.74 (0.29)<br>EBM: -0.22; 0.86 (0.33) |
| $k_{PABAK}$ | 0.54 (without paradoxes) | 0.29; 0.79 [19] | BM: 0.22; 0.87 (0.55)<br>EBM: 0.27; 0.77 (0.54) |
| $H$ | 0.77 (without paradoxes) | 0.64; 0.90 [19] | BM: 0.73; 0.97 (0.88)<br>EBM: 0.64; 0.88 (0.77) |
| $\Gamma$<br>$J$ | 0.30 (with paradoxes) | 0.23; 0.36 [59] | BM: 0.07; 0.63 (0.31)<br>EBM: 0.07; 0.60 (0.31) |
| $B$ | 0.71 (without paradoxes) | N.C. | BM: 0.46; 0.91 (0.72)<br>EBM: 0.54; 0.87 (0.71) |
| $\Delta$ | 0.54 (without paradoxes) | $\Delta_I$= 0.30; 0.79<br>$\Delta_{II}$ = 0.32; 0.77<br>[24] | BM: 0.28; 0.83 (0.56)<br>EBM: 0.30; 0.79 (0.57) |
| $\gamma$ ($AC_1$) | 0.68 (without paradoxes) | 0.56; 0.79 [21] | BM: 0.33; 0.90 (0.68)<br>EBM: 0.44; 0.86 (0.67) |

I-IT2

| | | A2 | | |
|---|---|---|---|---|
| | | 0 | 1 | |
| A1 | 0 | **30** | **1** | 31 |
| | 1 | **3** | **1** | 4 |
| | | 33 | 2 | 35 |

Agreement table

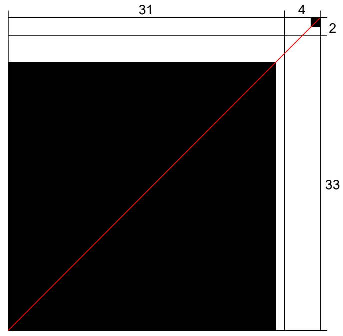


Observer agreement chart

| $P_o$ = 0.88 | | | |
|---|---|---|---|
| $\pi$ | 0.27 (with paradoxes) | -0.41; 0.95 [19] | BM: -012; 0.86 (0.26)<br>EBM: -0.11; 0.78 (0.23) |
| $k$<br>$\alpha$ | 0.27 (with paradoxes) | -0.40; 0.95 [62] | BM: -0.10; 0.78 (0.27)<br>EBM: -0.09; 0.78 (0.25) |
| $k_C$ | 0.43 (with paradoxes) | -0.05; 0.92 [62] | BM: -0.17; 1.00<br>EBM: N.C.[2] |
| $k_{PABAK}$ | 0.77 (without paradoxes) | 0.54; 0.98 [19] | BM: 0.45; 0.95 (0.76)<br>EBM: 0.54; 0.94 (0.77) |
| $H$ | 0.88 (without paradoxes) | 0.77; 0.99 [19] | BM: 0.74; 0.99 (0.88)<br>EBM: 0.77; 0.97 (0.89) |
| $\Gamma$<br>$J$ | 0.59 (with paradoxes) | 0.52; 0.67 [59] | BM: 0.28; 0.90 (0.59)<br>EBM: 0.29; 0.89 (0.61) |
| $B$ | 0.87 (without paradoxes) | N.C. | BM: 0.73; 0.97 (0.87)<br>EBM: 0.74; 0.97 (0.87) |
| $\Delta$ | 0.79 (without paradoxes) | $\Delta_I$= 0.58; 0.99<br>$\Delta_{II}$= 0.62; 0.96 [24] | BM: 0.52; 0.97 (0.79)<br>EBM: 0.57; 0.97 (0.81) |
| $\gamma\ (AC_1)$ | 0.86 (without paradoxes) | 0.75; 0.98 [21] | BM: 0.66; 0.99 (0.86)<br>EBM: 0.70; 0.97 (0.86) |

I-IT3

| | | 2 | | |
|---|---|---|---|---|
| | | 0 | 1 | |
| A1 | 0 | **42** | **2** | 44 |
| | 1 | **2** | **3** | 5 |
| | | 44 | 5 | 49 |

Agreement table

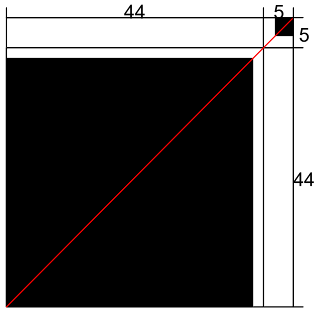


Observer agreement chart

| $P_o$ = 0.92 | | | |
|---|---|---|---|
| $\pi$ | 0.55 (with paradoxes) | 0.13; 0.98 [19] | BM: 0.17; 0.83 (0.54)<br>EBM: - 0.04; 0.90 (0.52) |
| $k$<br>$\alpha$ | 0.55 (with paradoxes) | 0.14; 0.97 [62] | BM: 0.21; 0.83 (0.55)<br>EBM: - 0.03; 0.90 (0.53) |
| $k_C$ | 0.55 (with paradoxes) | 0.55; 0.55 [62] | BM: 0.20; 1.00 (0.64)<br>EBM: -1.29; 5.09 (1.23) |
| $k_{PABAK}$ | 0.84 (without paradoxes) | 0.69; 0.99 [19] | BM: 0.71; 0.95 (0.84)<br>EBM: 0.67; 0.96 (0.84) |
| $H$ | 0.92 (without paradoxes) | 0.84; 0.99 [19] | BM: 0.86; 0.97 (0.92)<br>EBM: 0.80; 0.97 (0.90) |
| $\Gamma$<br>$J$ | 0.69 (with paradoxes) | 0.64; 0.76 [59] | BM: 0.51; 0.90 (0.71)<br>EBM: 0.45; 0.92 (0.70) |
| $B$ | 0.90 (without paradoxes) | N.C. | BM: 0.85; 0.98 (0.90)<br>EBM: 0.80; 098 (0.90) |
| $\Delta$ | 0.84 (without paradoxes) | $\Delta_I$= 0.68; 0.99<br>$\Delta_{II}$ = 0.70; 0.98 [24] | BM: 0.72; 0.97 (0.85)<br>EBM: 0.68; 0.98 (0.85) |
| $\gamma\ (AC_1)$ | 0.90 (without paradoxes) | 0.78; 1.02 [21] | BM: 0.81; 0.97(0.90)<br>EBM: 0.78; 0.98 (0.90) |

I-IT4

| | | A2 | | |
|---|---|---|---|---|
| | | 0 | 1 | |
| A1 | 0 | **33** | **2** | 35 |
| | 1 | **0** | **2** | 2 |
| | | 33 | 4 | 37 |

Agreement table

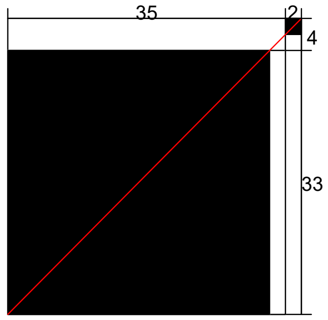


Observer agreement chart

| $P_o = 0.95$ | | | |
|---|---|---|---|
| $\pi$ | 0.64 (with paradoxes) | 0.14; 1.13 [19] | BM: 0.19; 0.92 (0.62)<br>EBM: - 0.04; 1.00 (0.59) |
| $k$<br>$\alpha$ | 0.64 (with paradoxes) | 0.16; 1.12 [62] | BM: 0.23; 0.93 (0.62)<br>EBM: N.C. |
| $k_C$ | 1 (with paradoxes) | 0.51; 1.48 [62] | BM: N.C.<br>EBM: -4.3; 8.33 |
| $k_{PABAK}$ | 0.89 (without paradoxes) | 0.76; 1.00 [19] | BM: 0.79; 0.97 (0.89)<br>EBM: 0.72; 1.00 (0.89) |
| $H$ | 0.95 (without paradoxes) | 0.88; 1.00 [19] | BM: 0.88; 0.99 (0.95)<br>EBM: 0.86; 1.00 (0.95) |
| $\Gamma$<br>$J$ | 0.79 (with paradoxes) | 0,72; 0,87 [59] | BM: 0.59; 0.95 (0.80)<br>EBM: 0.52; 1.00 (0.79) |
| $B$ | 0.94 (without paradoxes) | N.C. | BM: 0.88; 0.99 (0.94)<br>EBM: 0.85; 1.00 (0.94) |
| $\Delta$ | 0.95 (without paradoxes) | $\Delta_I$= 0.84; 1.05<br>$\Delta_{II}$= 0.84; 1.05<br>[24] | BM: 0.87; 0.99 (0.94)<br>EBM: 0.86; 1.00 (0.95) |
| $\gamma$ $(AC_1)$ | 0.94 (without paradoxes) | 0.82; 1.05 [21] | BM: 0.86; 0.99 (0.93)<br>EBM: 0.83; 1.00 (0.93) |

I-IT5

| | | A2 | | |
|---|---|---|---|---|
| | | 0 | 1 | |
| A1 | 0 | **38** | **2** | 40 |
| | 1 | **0** | **0** | 0 |
| | | 38 | 2 | 40 |

Agreement table

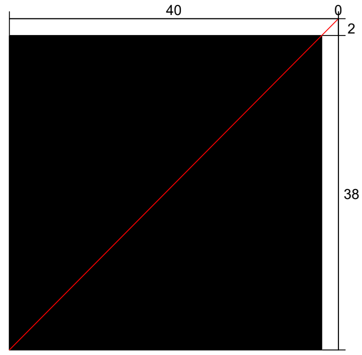


Observer agreement chart

| $P_o = 0.95$ | | | |
|---|---|---|---|
| $\pi$ | -0.02 (with paradoxes) | -1.43; 1.38 [19] | BM: N.C.<br>EBM: -0.07; 1.00 (0.10) |
| $k$<br>$\alpha$ | 0.00 (with paradoxes) | -1.35; 1.35 [62] | BM: N.C.<br>EBM: 0.00; 1.00 (0.13) |
| $k_C$ | 0.00 (with paradoxes) | N.C. | BM: N.C.<br>EBM: N.C. |
| $k_{PABAK}$ | 0.90 (without paradoxes) | 0.76; 1.00 [19] | BM: 074; 1.00 (0.90)<br>EBM: 0.75; 1.00 (0.90) |
| $H$ | 0.95 (without paradoxes) | 0.88; 1.00 [19] | BM: 0.86; 1.00 (0.95)<br>EBM: 0.87; 1.00 (0.95) |
| $\Gamma$<br>$J$ | 0.81 (with paradoxes) | 0.74; 0.88 [59] | BM: 0.51; 1.00 (0.79)<br>EBM: 0.56; 1.00 (0.81) |
| $B$ | 0.95 (without paradoxes) | N.C. | BM: 0.87; 1.00 (0.95)<br>EBM: 0.87; 1.00 (0.95) |
| $\Delta$ | 0.95 (without paradoxes) | N.C. | BM: 0.90; 1.00 (0.97)<br>EBM: 0.87; 1.00 (0.95) |
| $\gamma$ $(AC_1)$ | 0.95 (without paradoxes) | 0.83; 1.00 [21] | BM: 0.81; 1.00 (0.94)<br>EBM: 0.86; 1.00 (0.95) |

I-IT6

| | | A2 | | |
|---|---|---|---|---|
| | | 0 | 1 | |
| A1 | 0 | **27** | **1** | 28 |
| | 1 | **0** | **2** | 2 |
| | | 27 | 3 | 30 |

Agreement table

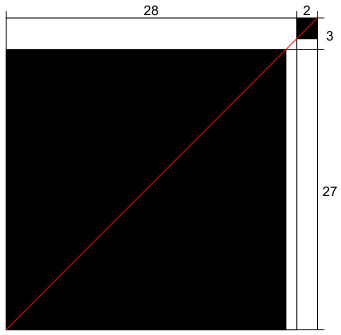


Observer agreement chart

| $P_o = 0.97$ | | | |
|---|---|---|---|
| $\pi$ | 0.78<br>(with paradoxes) | 0.35; 1.20<br>[19] | BM: 0.62; 0.94 (0.77)<br>EBM: 0.47; 0.80 (0.74) |
| $k$<br>$\alpha$ | 0.78<br>(with paradoxes) | 0.36; 1.20<br>[62] | BM: 0.00; 1.00 (0.77)<br>EBM: 0.00; 1.00 (0.74) |
| $k_C$ | 1.00<br>(with paradoxes) | 0.58; 1.41<br>[62] | BM: N.C.<br>EBM: N.C. |
| $k_{PABAK}$ | 0.93<br>(without paradoxes) | 0.82; 1.00<br>[19] | BM: 0.74; 1.00 (0.93)<br>EBM: 0.80; 1.00 (0.93) |
| $H$ | 0.97<br>(without paradoxes) | 0.91; 1.00<br>[19] | BM: 0.79; 0.99 (0.90)<br>EBM: 0.9; 1.00 (0.97) |
| $\Gamma$<br>$J$ | 0.87<br>(with paradoxes) | 0.78; 0.95<br>[59] | BM: 0.55; 1.00 (0.87)<br>EBM: 0.64; 1.00 (0.87) |
| $B$ | 0.96<br>(without paradoxes) | N.C. | BM: 0.88; 1.00 (0.96)<br>EBM: 0.88; 1.00 (0.96) |
| $\Delta$ | 0.97<br>(without paradoxes) | $\Delta_I$= 0.87; 1.00<br>$\Delta_{II}$ = 0.87; 1.00<br>[24] | BM: 0.87; 1.00 (0.97)<br>EBM: 0.90; 1.00 (0.97) |
| $\gamma\ (AC_1)$ | 0.96<br>(without paradoxes) | 0.84; 1.00<br>[21] | BM: 0.84; 1.00 (0.96)<br>EBM: 0.80; 1.00 (0.93) |

I-IT7

| | | A2 | | |
|---|---|---|---|---|
| | | 0 | 1 | |
| A1 | 0 | **29** | **1** | 30 |
| | 1 | **0** | **0** | 0 |
| | | 29 | 1 | 30 |

Agreement table

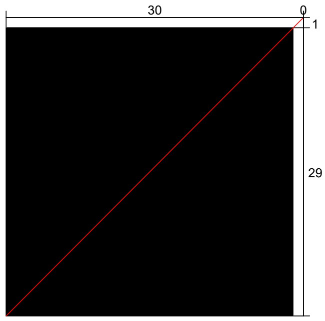


Observer agreement chart

| $P_o = 0.97$ | | | |
|---|---|---|---|
| $\pi$ | -0.02<br>(with paradoxes) | -2.01; 1.98<br>[19] | BM: N.C.<br>EBM: -0.05; 1.00 (0.35) |
| $k$<br>$\alpha$ | 0.00<br>(with paradoxes) | -1.93; 1.93<br>[62] | BM: N.C.<br>EBM: 0.00; 1.00 (0.36) |
| $k_C$ | 0.00<br>(with paradoxes) | N.C. | BM: N.C.<br>EBM: 0.00; 1.00 (0.00) |
| $k_{PABAK}$ | 0.93<br>(without paradoxes) | 0.82; 1.00<br>[19] | BM: 0.77; 1.00 (0.93)<br>EBM: 0.80; 1.00 (0.93) |
| $H$ | 0.97<br>(without paradoxes) | 0.91; 1.00<br>[19] | BM: 0.90; 1.00 (0.97)<br>EBM: 0.90; 1.00 (0.97) |
| $\Gamma$<br>$J$ | 0.87<br>(with paradoxes) | 0.78; 0.96<br>[59] | BM: 0.63; 1.00 (0.88)<br>EBM: 0.64; 1.00 (0.87) |
| $B$ | 0.97<br>(without paradoxes) | N.C. | BM: 0.91; 1.00 (0.97)<br>EBM: 0.90; 1.00 (0.97) |
| $\Delta$ | 0.97<br>(without paradoxes) | $\Delta_I$= 0.87; 1.00<br>$\Delta_{II}$= N.C.<br>[24] | BM: 0.88; 1.00 (0.97)<br>EBM: 0.90; 1.00 (0.97) |
| $\gamma\ (AC_1)$ | 0.96<br>(without paradoxes) | 0.85; 1.00<br>[21] | BM: 0.88; 1.00 (0.97)<br>EBM: 0.89; 1.00 (0.96) |

I-PT1

| | | 2 | | |
|---|---|---|---|---|
| | | 0 | 1 | |
| A1 | 0 | **40** | **0** | 40 |
| | 1 | **0** | **6** | 6 |
| | | 40 | 6 | 46 |

Agreement table

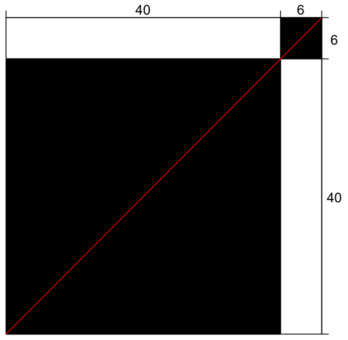


Observer agreement chart

| $P_o = 1.00$ | | | |
|---|---|---|---|
| $\pi$ | 1.00 (with paradoxes) | 1.00; 1.00 [19] | BM: 1.00; 1.00 (1.00)<br>EBM: 1.00; 1.00 (1.00) |
| $k$<br>$\alpha$ | 1.00 (with paradoxes) | 1.00; 1.00 [62] | BM: 1.00; 1.00 (1.00)<br>EBM: 1.00; 1.00 (1.00) |
| $k_C$ | 1.00 (with paradoxes) | -1.00; 1.00 [62] | BM: 1.00; 1.00 (1.00)<br>EBM: 1.00; 1.00 (1.00) |
| $k_{PABAK}$ | 1.00 (without paradoxes) | N.C. | BM: 1.00; 1.00 (1.00)<br>EBM: 1.00; 1.00 (1.00) |
| $H$ | 1.00 (without paradoxes) | N.C. | BM: 1.00; 1.00 (1.00)<br>EBM: 1.00; 1.00 (1.00) |
| $\Gamma$<br>$J$ | 1.00 (with paradoxes) | 0.94; 1.00 [59] | BM: 1.00; 1.00 (1.00)<br>EBM: 1.00; 1.00 (1.00) |
| $B$ | 1.00 (without paradoxes) | N.C. | BM: 1.00; 1.00 (1.00)<br>EBM: 1.00; 1.00 (1.00) |
| $\Delta$ | 1.00 (without paradoxes) | $\Delta_I$ = 1.00<br>$\Delta_{II}$= 1.00<br>[24] | BM: 1.00; 1.00 (1.00)<br>EBM: 1.00; 1.00 (1.00) |
| $\gamma\ (AC_1)$ | 1.00 (without paradoxes) | 0.88; 1.12 [21] | BM: 1.00; 1.00 (1.00)<br>EBM: 1.00; 1.00 (1.00) |

## References


63. Agresti, A. *Categorical Data Analysis*, 2nd ed.; Wiley: New York, NY, USA, 2002; pp. 1–356.

**Table S2.** Agreement table.

Observations can be organized in a contingency 2 (rater) × 2 (categories) table, in which the *N* objects are evaluated by a categorical scale of 2 categories. This table is known as agreement table [63].

| | | Rater B $q$ | | |
|---|---|---|---|---|
| | | 1 | 2 | Total |
| Rater A $q$ | 1 | $n_{11}$ | $n_{12}$ | $n_{1.}$ |
| | 2 | $n_{21}$ | $n_{22}$ | $n_{2.}$ |
| | Total | $n_{.1}$ | $n_{.2}$ | $n$ |

$q$ = categories; $n$ = subjects
